# A Backside-Electrode-Free Lateral 4H-SiC JFET with Three-Terminal Dual-Gate Design for Stable DC Operation at 500 °C

**DOI:** 10.3390/mi17060642

**Published:** 2026-05-22

**Authors:** Yuting Tang, Qian Luo, Jiang Zhu, Hezhi Zhang, Yuchun Chang, Hongwei Liang

**Affiliations:** 1 School of Integrated Circuits, Dalian University of Technology, Dalian 116024, China; tangyt@mail.dlut.edu.cn (Y.T.); zhuj@mail.dlut.edu.cn (J.Z.); hez.zhang@dlut.edu.cn (H.Z.); 2 Key Laboratory of Materials Modification by Laser, Ion, and Electron Beams (Ministry of Education), School of Physics, Dalian University of Technology, Dalian 116024, China; qianluo@mail.dlut.edu.cn

**Keywords:** high-temperature, 4H-SiC, JFET, TCAD

## Abstract

To address the urgent need for electronics operable in extremely high-temperature environments, this paper presents a novel three-terminal, dual-gate, lateral 4H-SiC n-channel depletion-mode junction field effect transistor (JFET) without a backside electrode. Featuring a fully planar electrode layout, the device eliminates the back-gate effect and significantly improves integration compatibility. Experimental results demonstrate stable DC operation up to 500 °C, with an intrinsic gain of 9.79 at room temperature and 6.01 at 500 °C. Comparison with TCAD simulations confirms excellent agreement in the key physical trends of threshold voltage drift and mobility degradation, though quantitative discrepancies are observed and attributed to process-induced parasitic effects such as non-ideal ohmic contacts and interface states. Analysis shows that the new structure broadens the channel depletion layer by optimizing the depletion profile, thereby suppressing channel-length modulation and improving both output resistance and gate control. This work not only provides an effective device platform for high-temperature 4H-SiC analog integrated circuits (ICs) but also deepens the understanding of process-performance correlations, offering clear guidance for process-oriented device optimization. The proposed structure serves as a foundation for developing fully planar, high-temperature 4H-SiC analog ICs with promising potential in aerospace, automotive, and energy exploration systems.

## 1. Introduction

As is well known, the integrated circuit industry has driven extensive research and development of silicon (Si) materials. However, in applications such as aerospace, metallurgy, chemical processing, oil and natural gas exploration, medical systems, high-speed rail, and automotive electronics, the operating environment often exceeds 300 °C [1,2,3,4]. Under such conditions, Si-based devices face limitations in maintaining normal performance. The failure of silicon-based electronic devices at high temperatures arises from fundamental material limitations, such as thermal instability of carrier density due to increasing intrinsic carrier concentration and the risk of dielectric breakdown [5]. However, wide-bandgap semiconductors show promise in overcoming the performance limitations of silicon. Among them, silicon carbide (SiC), gallium nitride (GaN), and aluminum nitride (AlN) have attracted extensive research interest. GaN suffers from high-temperature mobility degradation of its two-dimensional electron gas due to phonon scattering, lowering its drive current [6]. AlN, though promising for high-temperature and high-power applications, is hindered by challenges in film growth and stable doping [7]. In contrast, 4H-SiC possesses distinct advantages over many wide-bandgap semiconductors in these regards, further reinforced by the minimal temperature dependence of its bandgap even at temperatures exceeding 850 °C [8]. As the cornerstone of high-temperature integrated circuits, the exceptional thermal stability of SiC-based devices, such as SiC JFETs, plays a vital role in enhancing the overall stability of circuit performance across a wide temperature range [9,10,11]. A key advantage of the JFET structure is that it does not rely on a gate oxide layer. While SiC MOSFETs utilize a highly conductive SiO_2_/SiC interface, this interface is accompanied by challenges, including the presence of fixed positive charges and interface trap density. The absence of gate oxide makes JFETs particularly suitable for high-reliability and high-temperature applications. Furthermore, they exhibit a significantly lower on-resistance (R_ds,on_) compared to conventional silicon depletion-mode MOSFET(DMOS) devices [12,13].

Advances in materials and device fabrication technology have led to the development of numerous optimized SiC JFET structures. For example, a novel lateral JFET based on 4H-SiC has been proposed, featuring an optimized reduced surface field design for power applications that has been thoroughly investigated and successfully implemented [14]. The article further highlights the advantages of the vertical-channel lateral JFET (VC-LJFET) technology in 4H-SiC, such as reduced output capacitance and the capability to adjust threshold voltages at the mask design stage. Finally, the study presents a monolithic power integrated circuit that combines a high-performance lateral JFET with a low-voltage buffer, demonstrating successful integration. The advancement of VC-LJFET technology is expected to accelerate the adoption of system-on-chip solutions in harsh-environment applications. Moreover, demonstrating a key advancement, researchers have reported a two-level interconnect 4H-SiC JFET IC. The approximately 1 µm vertical structure successfully integrates hafnium ohmic contacts, TaSi_2_ interconnects, and SiO_2_/Si_3_N_4_ dielectrics. These innovations are pivotal for ensuring the long-term operational stability of ICs in high-temperature air environments up to 500 °C [15]. However, implementing backside contacts requires a diffusion barrier metallization stack (such as TaSi_2_/Pt/Ir/Pt) to prevent the interdiffusion of gold from bonding pads and atmospheric oxygen into the ohmic contact/SiC interface. Without such a barrier, high-temperature operation would lead to failure of the backside contact. Moreover, the long-term reliability at elevated temperatures is compromised between the diffusion barrier and the underlying ohmic metallization [16,17,18,19,20]. Adopting a front-side structure for the back electrode alleviates the challenges associated with high-temperature packaging and enhances integration compatibility. A systematic comparison of our device with the state-of-the-art SiC JFETs reported in Refs. [14,15,16,17,18,19,20] is presented in Table 1, highlighting the distinct advantages of our backside-electrode-free three-terminal dual-gate design. Specifically, the proposed front-side-only design eliminates wafer thinning, backside alignment, diffusion-barrier deposition, backside ohmic annealing, and special die-attach precautions required for conventional backside-contact JFETs, thereby simplifying both fabrication and high-temperature packaging. Beyond device technology, recent advances in 4H-SiC pressure sensors demonstrate the growing maturity of SiC for extreme-environment sensing. For instance, high-sensitivity 4H-SiC MEMS pressure sensors operating over wide temperature ranges [21], as well as leadless-packaged heavily doped n-type 4H-SiC pressure sensor families, have been reported [22]. These developments show that SiC sensing devices are moving toward practical high-temperature applications, thereby strengthening the motivation for developing fully planar, backside-electrode-free 4H-SiC JFETs as potential readout or signal-conditioning elements for future monolithically integrated SiC sensor-circuit systems.

In this work, we present the design, fabrication, and characterization of a novel three-terminal, dual-gate, lateral n-channel depletion-mode 4H-SiC JFET with a gate length of 10 μm and a gate width of 300 μm. Device performance was evaluated through a combination of numerical simulation and electrical measurements. The device exhibits an intrinsic gain of 9.79 at room temperature, which remains at 6.01 even at a high temperature of 500 °C. Compared with conventional designs, the proposed three-terminal dual-gate lateral configuration yields an optimized depletion profile, while the fully planar electrode layout eliminates back-gate effects and enhances compatibility with integrated circuit manufacturing. TCAD simulations show that performance improvement is intrinsically linked to a more uniform electric field distribution and a widened depletion layer in the channel, which suppresses channel-length modulation and improves gate-control linearity. Through measurements of the transfer and output characteristics of fabricated devices, the temperature dependence of key parameters such as threshold voltage, transconductance, and output resistance was systematically evaluated. A detailed comparison between experimental data and TCAD simulations reveals quantitative discrepancies in parameters such as threshold voltage and saturation current, while confirming excellent qualitative agreement in all major temperature-related trends, including the negative shift in threshold voltage and the degradation of saturation current. The observed discrepancies are attributed to inherent complexities in practical fabrication that challenge precise modeling. These include differences between the ideal simulated doping profile and the actual post-implantation defect distribution, as well as parasitic effects such as non-ideal ohmic contact resistance and interface traps that are inevitable in experiments but often simplified in simulations. Despite the quantitative differences, TCAD simulation remains fundamental in this work, having correctly identified all key performance trends and guided the initial structural optimization. This study offers a practical device-level solution and validates a structural-innovation approach to improving the intrinsic performance of 4H-SiC JFETs. The proposed structure provides a foundation for developing fully planar, high-temperature 4H-SiC analog integrated circuits, with promising potential for aerospace, automotive, and energy exploration systems.

## 2. Device Simulation and Design

### 2.1. Physical Models for High-Temperature Simulation in Sentaurus TCAD

To accurately simulate the device behavior from room temperature to 800 K, we employed the following physical models available in TCAD Sentaurus 0-2018.06-SP2.

Mobility Models: The Masetti model for doping-dependent mobility accounts for the reduction in carrier mobility due to impurity scattering at high doping concentrations. The model parameters in Sentaurus are temperature-scaled to ensure accurate mobility prediction across the entire temperature range. The Canali model for high-field saturation describes the velocity saturation effect at high electric fields, with temperature-dependent saturation velocity. This is particularly important for accurate current modeling under high drain bias at elevated temperatures.

Incomplete Ionization Model: Due to the relatively deep dopant levels in 4H-SiC, incomplete ionization significantly affects carrier concentration, especially at room temperature. We activated the incomplete ionization model in Sentaurus, which calculates the ionized dopant density as a function of temperature. This model is critical for correctly predicting the threshold voltage and its temperature dependence up to 800 K, where ionization approaches completion.

Recombination Model: Shockley-Read-Hall (SRH) recombination was enabled with temperature-dependent carrier lifetimes. The lifetime increases with temperature according to a power-law exponent typical for 4H-SiC, accounting for the enhanced recombination at elevated temperatures.

Thermal Models: The thermodynamic model is used to account for its own heat generation effect. This model solves the lattice heat flow equation alongside the drift-diffusion equations, capturing the local temperature rise due to power dissipation.

To address concerns regarding model validity at 800 K, we emphasize that all physical models employed in this study are standard Sentaurus TCAD models with built-in temperature-dependent parameters specifically calibrated for 4H-SiC. These models, including the Masetti mobility model, Canali model, incomplete ionization model and SRH recombination, are part of Sentaurus’s validated material database for silicon carbide. The temperature dependencies incorporated in these models are based on fundamental physical principles (phonon scattering, dopant ionization thermodynamics, etc.) that remain valid across the entire temperature range.

### 2.2. Analysis of Simulation Results

**Figure 1:**
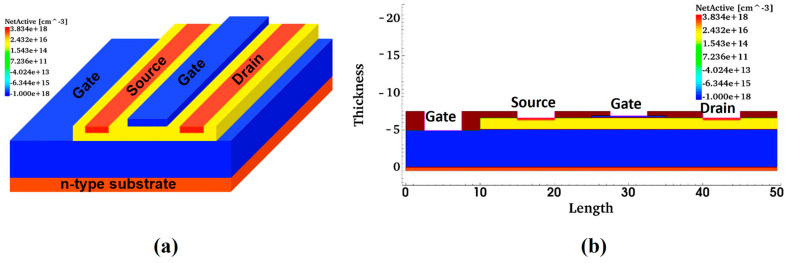
(**a**) 3-D rendition of actual device; (**b**) TCAD 2-D cross section.

Figure 1a shows a 3-D structure of a 4H-SiC JFET. The device under study has a gate length of 10 µm and a gate width of 300 μm. The device simulation structure is an ordered epitaxial stack grown on an n-type substrate (Powerepi Semiconductor, Guangzhou, China). The first epitaxial layer is p-type with a thickness of 5 µm and a doping concentration of 1 × 10^18^ cm^−3^. The second epitaxial layer is n-type with a thickness of 1.7 µm and a doping concentration of 1 × 10^16^ cm^−3^. The third epitaxial layer is p-type with a thickness of 0.2 µm and a doping concentration of 1 × 10^18^ cm^−3^.

The 2-D cross-section of the fabricated device was generated using the TCAD Sentaurus Process tool and subsequently simulated with the TCAD Sentaurus Device for numerical analysis. Figure 1b shows the 2-D section used in the TCAD Sentaurus Process tool, and the device performance and mechanism are simulated through TCAD. The nitrogen N^+^ source/drain implant was obtained through Monte Carlo simulation [13,23,24]. The length and thickness of ion implantation in the source region and drain region are 5 µm and 0.4 µm, respectively, according to the Monte Carlo method. The implantation process was designed to yield a doping profile with a high concentration of 3.8 × 10^18^ cm^−3^ near the surface, so that the lightly doped layer can form low resistance ohmic contacts. Adaptive meshing was performed on the structure, and subsequent device simulation using TCAD Sentaurus Device was conducted to extract its electrical characteristics. The simulation employs the drift-diffusion transport model, solving the coupled system of the Poisson equation, carrier continuity equations, and current density equations for electrons and holes. As a result, the simulation incorporates key thermal effects, including self-heating, lattice temperature rise, and carrier heating. This process yields key electrical performance parameters under given conditions, directly guiding device optimization.

**Figure 2:**
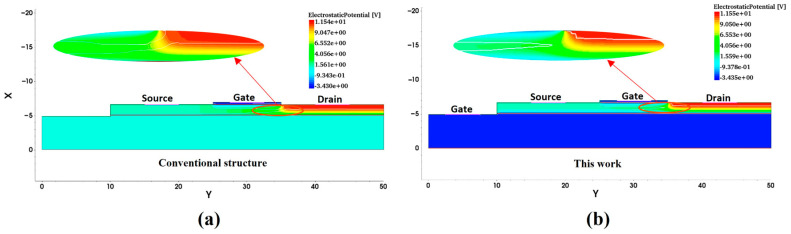
Electrostatic potential distributions in (**a**) a conventional lateral 4H-SiC JFET; (**b**) the proposed three-terminal dual-gate lateral 4H-SiC JFET.

In the TCAD simulations, the overall device structure was maintained constant between the traditional and the proposed three-terminal dual-gate lateral 4H-SiC JFETs, thereby ensuring a one-to-one comparison of their performance. Figure 2 illustrates the two-dimensional electrostatic potential distribution within the device under output characteristic simulation, clearly revealing the potential gradient from source to drain. In the conventional lateral 4H-SiC JFET, the n-channel equipotential lines inside the marked red circle remain connected, indicating that the single gate cannot fully deplete the channel, making complete pinch-off difficult to achieve. By contrast, the proposed three-terminal dual-gate lateral 4H-SiC JFET exhibits enhanced gate control owing to the combined action of the upper and lower gates. As highlighted in the corresponding red circle, the spacing between equipotential lines in the n-channel is visibly wider, reflecting a noticeable reduction in the electric-field peak. The peak voltage of the conventional lateral structure reaches 8.241 V, while that of the three-terminal dual-gate lateral design reaches 6.553 V, a decrease of 20.4%. This improvement stems from the three-terminal dual-gate design, which optimizes the electrode layout and reshapes the depletion region, thereby mitigating local electric-field crowding. In the conventional design, the electric field concentrates at the drain-side gate edge due to the steep potential gradient. The dual-gate configuration alleviates this by introducing an additional gate that screens the drain potential and distributes the voltage drop more uniformly along the channel. This field-smoothing effect, evidenced by the 20.4% reduction in peak field, is expected to translate into improved breakdown voltage and enhanced reliability, although experimental breakdown validation remains for future work.

**Figure 3:**
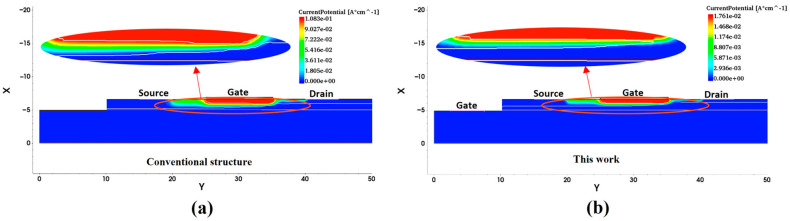
Current density distribution in (**a**) a conventional lateral 4H-SiC JFET; (**b**) the proposed three-terminal dual-gate lateral 4H-SiC JFET.

Figure 3 presents a comparative analysis of the current density distribution for two device architectures. In the conventional lateral structure, the current density is highly localized, reaching a peak of 1.083 × 10^−1^ A·cm^−1^, indicating pronounced current crowding, which can lead to localized heating and reliability issues. In contrast, the three-terminal dual-gate lateral design exhibits a markedly lower and more uniformly distributed current density, with a peak value of only 1.761 × 10^−2^ A·cm^−1^, a decrease of 83.7%. The widened and smoothed distribution in Figure 3b correlates with the previously observed more uniform electric field, confirming that the optimized electrode design successfully mitigates carrier accumulation in critical regions. This improvement is crucial for enhancing the device’s safe operating area and long-term reliability under high- temperature conditions.

**Figure 4:**
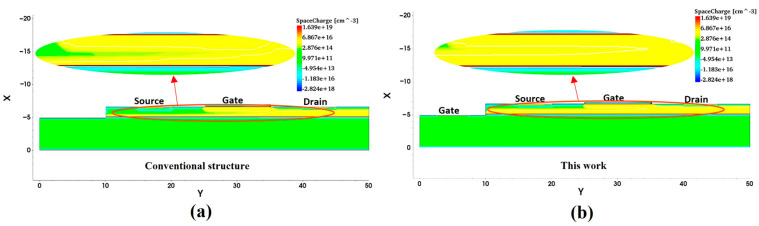
Space charge distribution in (**a**) a conventional lateral 4H-SiC JFET; (**b**) the proposed three-terminal dual-gate lateral 4H-SiC JFET.

Figure 4 compares the two-dimensional space charge density distributions under typical bias conditions for a conventional lateral 4H-SiC JFET and the proposed three-terminal dual-gate structure in this work. The color gradient, ranging from high positive charge (1.639 × 10^19^ cm^−3^) to high negative charge density (−2.824 × 10^18^ cm^−3^), clearly delineates the spatial profile of the depletion regions. In the conventional structure, the depletion region beneath the gate exhibits limited and asymmetric extension into the n-channel. In contrast, in the three-terminal dual-gate structure, the coordinated action of the upper and lower gates results in a more extended, symmetric, and uniform depletion layer within the n-channel. This demonstrates that the depletion layer penetrates more deeply and comprehensively into the channel. This enhanced depletion effect, originating from the additional gate control provided by the three-terminal dual-gate design, enables more effective modulation of channel carriers. The comparison, particularly from the perspective of depletion layer widening, confirms that the three-terminal dual-gate structure achieves more complete and uniform channel depletion. This improvement not only helps alleviate electric field peaking but also enhances gate control capability and threshold voltage stability. Consequently, it provides a critical structural advantage for reliable device operation under high-temperature and high-power conditions.

**Figure 5:**
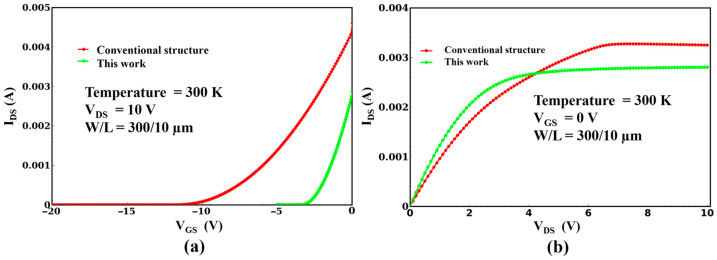
Simulated results (**a**) transfer characteristic curve; (**b**) output characteristic curve for the conventional lateral 4H-SiC JFET and the proposed three-terminal dual-gate lateral structure.

Figure 5 compares the electrical characteristics of the traditional structure and the three-terminal dual-gate lateral 4H-SiC JFET proposed in this study at room temperature (300 K), revealing the performance characteristics and design trade-offs between the two. Figure 5a shows the transfer characteristic curve. At V_DS_ = 10 V, the drain current of the traditional structure is slightly higher than that of the three-terminal structure in this work. However, it is worth noting that the curve of the three-terminal dual-gate structure has a steeper slope in the proximity of the threshold region. This indicates that the three-terminal dual-gate structure exhibits a more sensitive and efficient control of the gate voltage on the channel current. This improved gate control capability stems from the synergy of the upper and lower gates, achieving more precise modulation of the channel potential. Figure 5b shows the output characteristic comparison, revealing more significant differences. At V_GS_ = 0 V, the saturation region curve of the traditional structure shows a significant upward curvature and poor flatness, which is a typical manifestation of the strong channel length modulation effect, indicating a lower output resistance. In contrast, the curve of the three-terminal dual-gate structure is significantly flatter in the saturation region, indicating a higher output resistance. This is directly attributed to the optimized depletion region shape of the new structure, which effectively suppresses the modulation of the channel length by drain-induced channel modulation, thereby improving the constant current characteristics of the device. This difference in electrical performance is consistent with the phenomenon observed in the previous simulation. The three-terminal dual-gate structure has a more uniform electric field distribution and a more fully expanded depletion layer, meaning it has a higher resistance output. This is due to the optimized depletion region that weakens the invasion of the leakage electric field into the channel, thereby improving the output resistance and gate control linearity. Consequently, the three-terminal dual-gate design sacrifices some peak current in exchange for superior comprehensive performance in terms of linearity and high-temperature stability, making it more suitable for applications in high-reliability analog integrated circuits.

**Figure 6:**
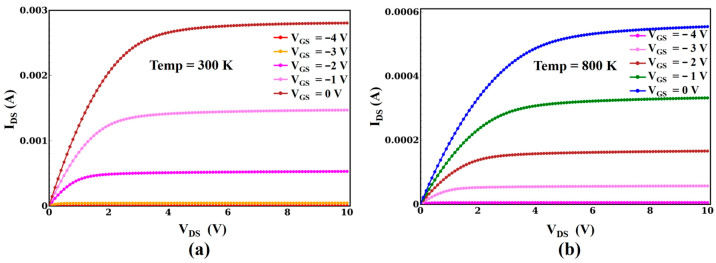
Simulated results (**a**) output characteristic curve at 300 K; (**b**) output characteristic curve at 800 K.

Figure 6 presents the simulated output characteristics of the proposed three-terminal dual-gate lateral 4H-SiC JFET at 300 K and 800 K. At room temperature, the device exhibits well-behaved transistor operation with distinct linear and saturation regions, confirming effective gate control. The most prominent change at 800 K is the significant reduction in the saturation current level. This is primarily caused by carrier mobility degradation with increasing temperature. Importantly, despite the reduction in current, the characteristic curves fully retain their fundamental shape, particularly the clear saturation behavior. This indicates that even at this extreme temperature, the gate retains effective control over the channel, and the core transistor functionality does not degrade. This simulation theoretically predicts the device’s operational capability at 800 K. This provides a critical performance expectation for stable device operation at 500 °C and beyond.

**Figure 7:**
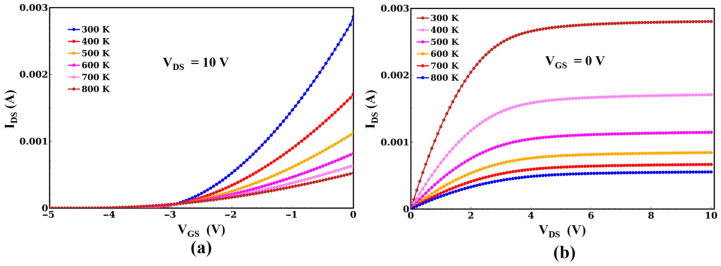
Simulated results (**a**) transfer characteristic curve; (**b**) output characteristic curve under different temperatures.

Figure 7 presents the temperature-dependent electrical characteristics of the proposed three-terminal dual-gate lateral 4H-SiC JFET. The transfer characteristics in Figure 7a show a clear negative shift in the threshold voltage (V_TH_) as temperature increases, accompanied by an overall reduction in the saturation current level under the same gate bias. These trends are further confirmed by the output characteristics in Figure 7b, which show a consistent decrease in saturation current at elevated temperatures. The observed behavior arises from fundamental semiconductor physics. The negative shift of V_TH_ is primarily attributed to the decrease in built-in potential with temperature, which facilitates the formation of the depletion region. Simultaneously, the decline in current stems from the degradation of carrier mobility due to enhanced phonon scattering at higher temperatures. Despite these parameter variations, the simulation confirms that the device maintains well-defined transfer and output saturation characteristics across the entire temperature range up to 800 K, with no indication of characteristic collapse or functional failure. This demonstrates the inherent robustness of the proposed three-terminal dual-gate structure under high-temperature conditions. The simulation results provide guidance for experimental device fabrication.

## 3. Device Fabrication

**Figure 8:**
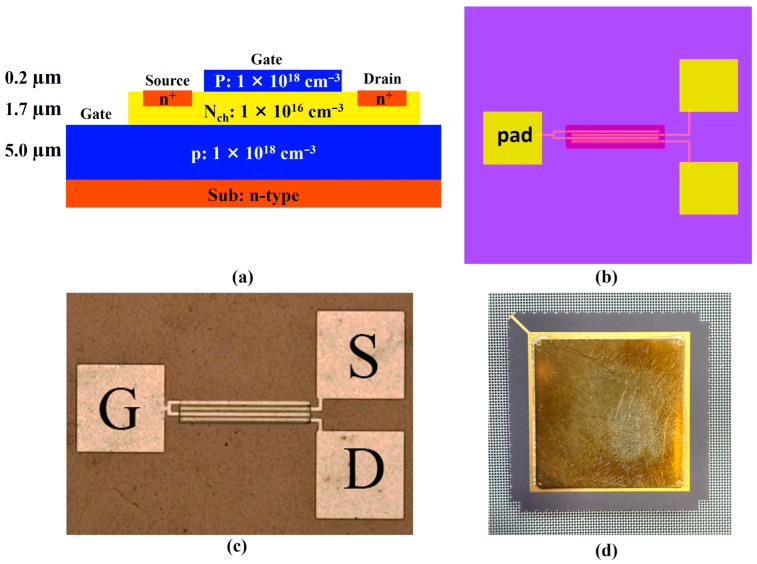
(**a**) Schematic cross section of gate with L = 10 µm and W = 300 µm; (**b**) Schematic of the sensor lithography layout; (**c**) optical micrograph of the fabricated 4H-SiC JFET device; (**d**) Optical image of the packaged device.

Figure 8a presents a schematic cross-sectional view of the fabricated 4H-SiC lateral JFET structure. The device was fabricated using a custom n-type 4H-SiC substrate with three additional epitaxial layers. The substrate doping concentration is 1 × 10^18^ cm^−3^. Figure 8b displays the photolithography layout. Photolithography was performed using equipment from Durham Magneto Optics Ltd. (Durham, UK). Device isolation was achieved through deep trench etching via inductively coupled plasma (ICP) etching (ICP-5100 system), with a trench depth of 7 µm. Subsequently, the gate region was etched to a depth of 2 µm, followed by source and drain recess etching of 0.3 µm, also performed using ICP etching. During this process, a nickel (Ni) metal (Powerepi Semiconductor, Guangzhou, China) mask of 300 nm thickness was used. Following ICP etching, a sacrificial oxide layer was grown at 1100 °C in dry O_2_ for 30 min and subsequently removed by diluted HF etching. This step consumes approximately 50 nm of the damaged sidewall, effectively removing the most defective layer and leaving a cleaner, more ordered interface. After sacrificial oxidation, the wafers were subjected to a high-temperature annealing step at 1300 °C in Ar ambient for 60 min. This annealing promotes the reconstruction of remaining lattice damage and reduces the density of interface traps. To form ohmic contacts for the source and drain, nitrogen (N) ion implantation was conducted at a 0° tilt angle to create n^+^ contact regions. Implantation doses were administered in four steps: 1.6 × 10^13^ cm^−2^, 1.8 × 10^13^ cm^−2^, 2.0 × 10^13^ cm^−2^, and 1.8 × 10^13^ cm^−2^. Post-implantation annealing was carried out at 1700 °C for 30 min under a protective carbon cap. A 1 μm-thick silicon dioxide (SiO_2_) (Powerepi Semiconductor, Guangzhou, China) layer was then deposited by magnetron sputtering. After opening contact windows in the passivation layer, Ni (10 nm)/Ti (10 nm)/Al (80 nm) (Powerepi Semiconductor, Guangzhou, China) stacks were deposited on the gate via electron beam evaporation, while Al (50 nm)/Ti (25 nm)/Au (50 nm) stacks were deposited on the source and drain. Ohmic contact formation was achieved through annealing at 900 °C for 5 min and at 830 °C for 5 min, both in a nitrogen (N_2_) atmosphere. A 1 μm-thick aluminum layer was subsequently deposited and patterned by magnetron sputtering to form the interconnects. Finally, a 1 µm-thick aluminum pad layer was patterned to complete the contact pads. Magnetron sputtering and electron beam evaporation were carried out using equipment manufactured by Shenyang Scientific Instrument Co., Ltd. (Shenyang, China).

Figure 8c shows an optical micrograph of the 4H-SiC lateral JFET (prior to packaging), with a gate length L = 10 µm and gate width W = 300 µm. Figure 8d shows the packaged device, which was subsequently subjected to electrical characterization. Electrical characterization was performed using a Keysight B1505A semiconductor parameter analyzer (Keysight Technologies, Santa Rosa, CA, USA). The experimental results presented in this work were obtained from devices with a fixed channel length of 10 µm, chosen to minimize short-channel effects and to establish the baseline behavior of the dual-gate design. For practical IC applications, understanding device scalability is essential. While experimental data for scaled devices is not yet available, the dual-gate structure offers inherent advantages for channel-length scaling. The enhanced electrostatic control provided by two adjacent gates is expected to suppress short-channel effects such as threshold voltage roll-off and drain-induced barrier lowering (DIBL), thereby maintaining good output resistance even at reduced channel lengths. Based on device physics principles and general trends for similar SiC JFETs, we anticipate that the proposed design can be scaled to channel lengths of 1–2 µm without catastrophic performance degradation. Further scaling below 1 µm would require optimization of doping profiles and contact metallization, as well as advanced lithography. Experimental validation of scaling behavior remains an important direction for future work.

## 4. Results and Discussion

**Figure 9:**
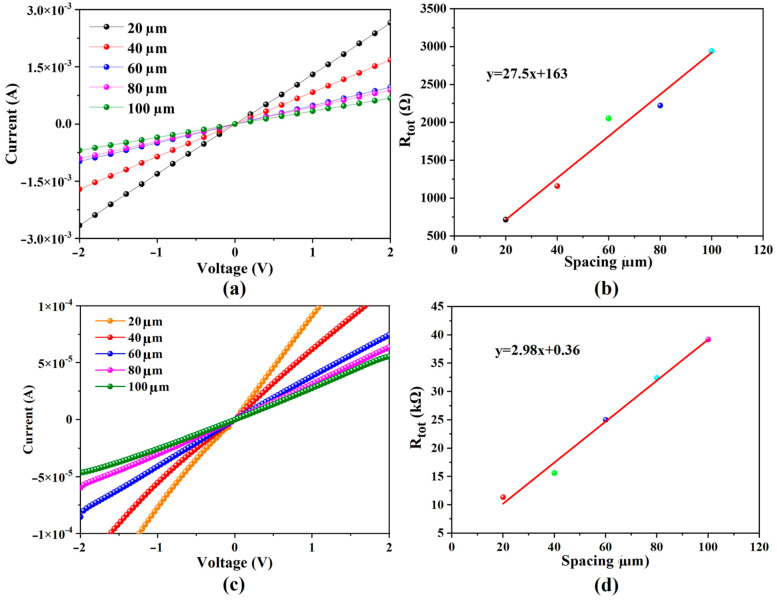
On-chip TLM characterization at 25 °C: (**a**) gate p-type; (**b**) TLM fitting curves; (**c**) source/drain n-type ohmic contact characteristics; (**d**) TLM fitting curves.

Ohmic contacts are critical for achieving high-performance 4H-SiC JFETs. In this work, the electrical characteristics of the contacts were evaluated using a transmission line measurement (TLM) structure. Figure 9a,b presents the current-voltage (I-V) characteristics of the p-type gate Ohmic contact, along with the corresponding TLM fitting curve. The specific contact resistivity (ρ_c_) and sheet resistance (R_sh_) of the p-type gate contact were determined to be 4.82 × 10^−4^ Ω·cm^2^ and 5.52 kΩ/sq, respectively. Meanwhile, Figure 9c,d shows the I-V characteristics of the n-type source/drain Ohmic contact, which exhibited significantly lower values of ρ_c_ = 1.23 × 10^−5^ kΩ·cm^2^ and R_sh_ = 72.40 kΩ/sq.

**Figure 10:**
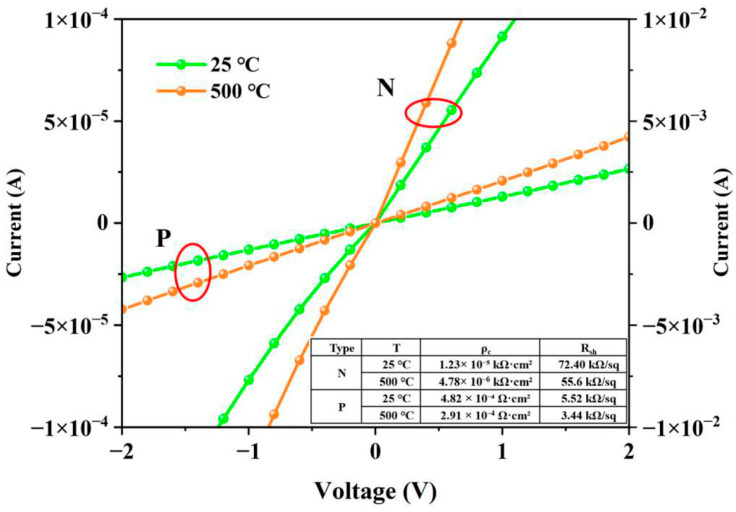
The N-type and P-type I-V curves with spacing d = 20 µm and extracted TLM parameters (inset) at 25 °C and 500 °C.

As shown in Figure 10, both n-type and p-type contacts maintain good ohmic contact characteristics at 500 °C. At elevated temperatures, both ρ_c_ and R_sh_ decrease. The reduction in R_sh_ indicates that the increase in free carrier concentration due to more complete ionization of dopants outweighs the mobility degradation caused by enhanced phonon scattering. The reduction in ρ_c_ is attributed to the increased average kinetic energy of carriers.

**Figure 11:**
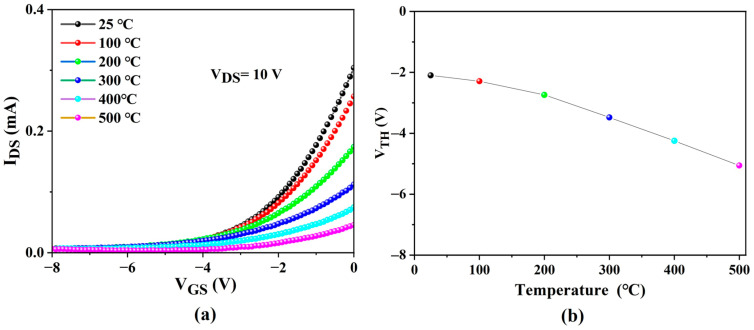
(**a**) Measured transfer characteristics curves (I_DS_-V_GS_) of the fabricated device at V_DS_ = 10 V and at various temperatures; (**b**) Threshold voltage extracted at different temperatures.

The transfer characteristics of the device, showing the relationship between drain-source current (I_DS_) and gate-source voltage (V_GS_) at various temperatures, are shown in Figure 11a. The measurements were performed under a fixed drain-source bias of V_DS_ = 10 V. The extracted threshold voltages are summarized in Figure 11b. At 25 °C, the V_TH_ was measured to be −2.1 V. The negative shift in threshold voltage with temperature is attributed to multiple factors, including the decrease in built-in potential, the slight increase in intrinsic carrier concentration, and the corresponding change in effective channel thickness. However, the relative contribution of these mechanisms is strongly dependent on the semiconductor material properties. For 4H-SiC, the wide bandgap (Eg = 3.26 eV) ensures that while the intrinsic carrier concentration does increase with temperature, its effect on threshold voltage remains negligible. The observed V_TH_ shift is therefore dominated by the temperature dependence of the built-in potential, which decreases with temperature due to bandgap narrowing and Fermi level movement toward the midgap. In contrast, narrow-bandgap materials such as silicon and gallium arsenide lose their gate control capability at high temperatures. Their lower thermal conductivity and material degradation at high temperatures further limit their applicability in extreme environments. Therefore, 4H-SiC offers superior high-temperature stability, and the predictable variation of V_TH_ is beneficial for circuit design.

**Figure 12:**
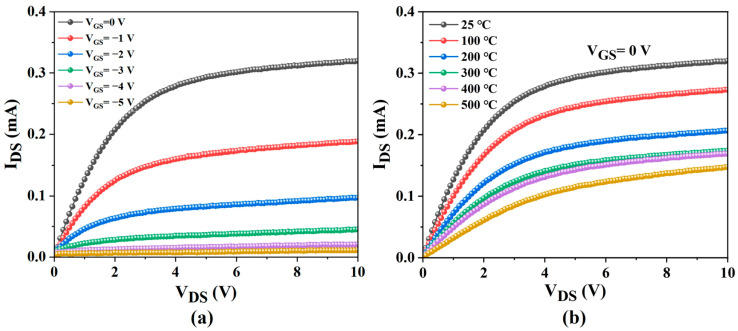
Typical measured drain output characteristics (I_DS_-V_DS_) curves (**a**) at 25 °C, and (**b**) for V_GS_ = 0 V at different temperatures of the fabricated 4H-SiC JFET with L = 10 µm and W = 300 µm.

Figure 12a shows the output characteristics of the fabricated 4H-SiC JFET at room temperature, illustrating the relationship between drain-source current and drain-source voltage. The gate-source voltage was swept from 0 V to −5 V in steps of −1 V. The results confirm typical transistor behavior of the fabricated device. At 25 °C, under a bias condition of V_DS_ = 10 V and V_GS_ = 0 V, the saturated drain-source current was measured to be 1.1 μA/μm. Figure 12b presents the output characteristics of the same device measured at temperatures ranging from room temperature up to 500 °C, in increments of 100 °C. The device maintained excellent transistor performance even at 500 °C. As the temperature increased, the saturated drain-source current (I_DS,SAT_) gradually decreased to 0.5 μA/μm at 500 °C. This reduction is primarily attributed to the decrease in electron mobility at elevated temperatures, which is consistent with the typical thermal behavior of JFETs in which the maximum drain current decreases with temperature. These findings confirm that the fabricated 4H-SiC JFET exhibits robust performance at elevated temperatures.

As shown in Table 2, the TCAD model predicts the threshold voltage with reasonable accuracy, showing deviations of 0.2 V at room temperature and 0.86 V at 500 °C. These deviations are within acceptable ranges for high-temperature device modeling and reflect the model’s capability to capture the temperature-dependent behavior of V_TH_. However, the saturation current shows substantial deviations: the simulated I_DS,SAT_ is 9.3 μA/μm compared to the measured 1.1 μA/μm at 25 °C and 1.6 μA/μm compared to 0.5 μA/μm at 500 °C. The observed differences are attributed to the inherent complexity of the actual manufacturing process, which poses a challenge for precise modeling. These complexities include the disparity between the doping distribution in the ideal simulation and the defect distribution after actual implantation, as well as inevitable parasitic effects such as non-ideal ohmic contact resistance and interface traps, which exist in experiments but are often simplified in simulations. To move beyond a purely qualitative explanation, we have used the TLM data in Figure 10 to quantitatively estimate the impact of ohmic contact resistance. Using the TLM-extracted n-type sheet resistance R_sh_ = 72.4 kΩ/sq at 25 °C (Figure 10), it is evident that the effective carrier concentration in the implanted source/drain regions is very low, leading to a high series resistance that severely limits the measured current. At 500 °C, R_sh_ decreases to 55.6 kΩ/sq, consistent with improved ionization and a reduced current overestimation. Contact resistance and interface traps contribute secondarily. Thus, the primary cause of the discrepancy is the much lower-than-expected activation efficiency in the implanted layers, which the ideal TCAD model does not capture.

For analog circuit design, key performance parameters include transconductance (g_m_), output resistance (r_o_), and intrinsic gain (g_m_r_o_). The corresponding parameters were extracted from Equations (1) and (2).

(1) $$g_{m}=\frac{\partial I_{DS}}{\partial V_{GS}}|V_{DS}=10\;V$$

(2) $$r_{o}=\frac{\partial V_{DS}}{\partial I_{DS}}|V_{GS}=0\;V$$

**Figure 13:**
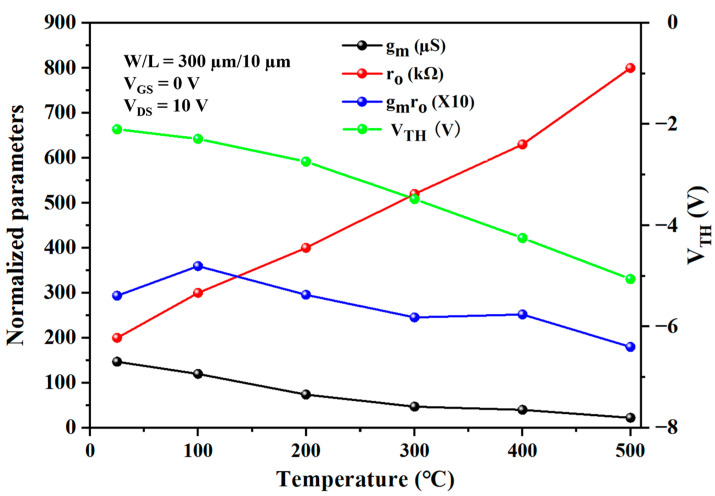
Temperature dependence of V_TH_, transconductance (g_m_), output resistance (r_o_) and intrinsic gain (g_m_r_o_) of JFET with L = 10 µm and W = 300 µm.

For the fabricated device, g_m_ and r_o_ were extracted at V_DS_ = 10 V and V_GS_ = 0 V using Equations (1) and (2). As shown in Figure 13, the device exhibits an output resistance of 2.02 × 10^6^ Ω, corresponding to an intrinsic gain of 9.79 at room temperature. Given that the intrinsic gain of short-channel devices in modern CMOS technologies typically ranges between 5 and 10 [25,26], this value is well suited for circuit applications. As the temperature increases to 500 °C, the r_o_ rises to 8.0 × 10^6^ Ω. This increase in r_o_ compensates for the degradation in g_m_ that occurs with temperature, thereby offsetting the reduction in g_m_. Consequently, the g_m_r_o_ experiences only a modest decrease to 6.01. At high temperature, the gain reduction is limited to about 38% for the three-terminal dual-gate structure, as opposed to about 63% for the conventional back-electrode device [27]. Nevertheless, comprehensive long-term reliability studies, including extended thermal cycling and bias stress testing, are necessary to fully validate the device for prolonged operation in harsh environments and are planned for future investigations. The experimental results presented here are based on a representative device. However, wafer-level variations in epitaxial layer thickness, doping uniformity, and processing conditions can lead to device-to-device differences. Despite these potential variations, measurements on three devices from different die locations confirmed consistent trends in threshold voltage temperature dependence and output characteristics, indicating that the observed dual-gate advantages are robust across the wafer. A full statistical study including device-to-device variation, yield, and error bars is planned for future work. These findings demonstrate that the 4H-SiC JFET developed in this study holds significant promise for high-temperature electronic applications.

## 5. Conclusions

In summary, this work presents the design, fabrication, and characterization of a novel three-terminal dual-gate n-channel depletion-mode 4H-SiC JFET fabricated without a backside electrode. The device demonstrates stable electrical operation under the tested DC conditions at temperatures up to 500 °C. Compared with conventional designs, the proposed three-terminal dual-gate lateral configuration achieves an optimized depletion profile, while the fully planar electrode layout eliminates back-gate effects and enhances compatibility with integrated circuit manufacturing. TCAD simulations indicate that the performance improvement originates from a more uniform electric-field distribution and a widened depletion layer in the channel, which suppresses channel-length modulation and improves gate control linearity. Through measurements of the transfer and output characteristics of fabricated devices, the temperature dependence of key parameters such as threshold voltage, transconductance, and output resistance was systematically evaluated. The intrinsic gain remains 6.01 at 500 °C, compared with 9.79 at room temperature. A detailed comparison between experimental data and TCAD simulations reveals quantitative discrepancies in parameters such as threshold voltage and saturation current, while confirming excellent qualitative agreement in all major temperature-related trends, including the negative shift of V_TH_ and the degradation of I_DS,SAT_. The observed discrepancies are attributed to inherent complexities in practical fabrication that challenge precise modeling. These include differences between the ideal simulated doping profile and the actual post-implantation defect distribution, as well as parasitic effects such as non-ideal ohmic contact resistance and interface traps that are inevitable in experiments but often simplified in simulations. Rigorous analysis of these differences provides deeper insight into how specific process variations quantitatively affect the electrical characteristics of 4H-SiC JFETs, moving beyond simple performance prediction toward a clearer understanding of process–device correlations. Despite the quantitative differences, TCAD simulation remains fundamental to this work, having correctly identified all key performance trends and guided the initial structural optimization. This study offers a practical device-level solution and validates a structural-innovation approach to improving the intrinsic performance of 4H-SiC JFETs. The proposed structure provides a foundation for developing fully planar, high-temperature 4H-SiC analog integrated circuits, with promising potential for aerospace, automotive, and energy exploration systems. Future work will focus on evaluating long-term reliability under extreme thermal stress and, based on these findings, implementing basic analog building blocks such as operational amplifiers using the present JFET technology to fully assess its circuit-level potential.

## Figures and Tables

**Table 1:** Comparison of the proposed dual-gate lateral JFET with previously reported SiC JFET structures.

Reference	Device Structure	Back Electrode	Gate Configuration	Key Operating Principle	Max Reported Temp.	Fabrication Complexity
[14]	Vertical-channel lateral JFET with double RESURF	No	Dual gates	Vertical channel formed by tilted implants on mesa sidewalls; double-RESURF for field shaping	300 °C	High
[15]	Lateral JFET with two-level interconnect	Yes	Single top gate	Hafnium ohmic contacts with TaSi_2_ interconnects; SiO_2_/Si_3_N_4_ dielectric layers	500 °C	High
[16]	Lateral epilayer JFET	Yes	Single top gate	Standard lateral JFET with body bias effect study	500 °C	Moderate
[17]	Lateral JFET with Ti contacts	Yes	Trenched gate	Buried gate structure with vertical channel	500 °C	Very high
[18]	Lateral JFET with Ti contacts	Yes	Single top gate	Body-biased through substrate; studied for SPICE modeling	961 °C	Moderate
[19]	Lateral JFET with two-level interconnect	Yes	Single top gate	TaSi_2_ interconnect	700 °C	High
[20]	Bondable metallization stack for JFET ICs	Yes	Single top gate	TaSi_2_/Pt/Ir/Pt diffusion barrier for high-temperature packaging	500 °C	High
This work	Lateral JFET with Three-Terminal	No	Dual top gates	Front-side dual-gate control, field redistribution, no backside processing	500 °C	Low

**Table 2:** Percentage deviation between TCAD simulations and experimental measurements for V_TH_ and I_DS,SAT_ at different temperatures.

Temperature	Parameter	Simulated Value	Experimental Value	Absolute Deviation
25 °C (300 K)	V_TH_	−2.30 V	−2.10 V	0.2 V
	I_DS,SAT_	9.3 μA/μm	1.1 μA/μm	8.2 μA/μm
500 °C (800 K)	V_TH_	−4.20 V	−5.06 V	0.86 V
	I_DS,SAT_	1.6 μA/μm	0.5 μA/μm	1.1 μA/μm

## Data Availability

Data are contained within the article.
